# Epidemiology of pediatric femur fractures in children: the Swedish Fracture Register

**DOI:** 10.1186/s12891-020-03796-z

**Published:** 2020-12-01

**Authors:** Zandra Engström, Olof Wolf, Yasmin D. Hailer

**Affiliations:** Section of Orthopaedics, Department of Surgical Sciences, Uppsala University, Uppsala, Sweden

**Keywords:** Femur, Fracture, Children, Epidemiology, Swedish FractureRegister

## Abstract

**Background:**

Although femur fractures in children are rare, they are the most common fractures in need of hospitalization. We sought to describe the epidemiology and treatment of pediatric femur fractures recorded in the Swedish Fracture Register (SFR). We also studied the relationship between femur fractures, age, sex, fracture pattern, injury mechanism, seasonal variation and treatment.

**Methods:**

This nationwide observational register study was based on the pediatric part of the SFR. We included all patients < 16 years of age who were registered in the SFR from 2015 to 2018.

**Results:**

Of the 709 femur fractures, 454 (64%) occurred in boys. Sixty-two of these fractures were proximal (9%), 453 shaft (64%) and 194 distal (27%). A bimodal age distribution peak was observed in boys aged 2–3 and 16–19 years. In contrast, the age distribution among girls was evenly distributed. Younger children were mainly injured by a fall, whereas older children sustained their fracture because of traffic accidents. Non-surgical treatment prevailed among younger children; however, prevalence of surgical treatment increased with age.

**Conclusions:**

We found a lower ratio between boys and girls (1.8:1) compared to earlier studies. The bimodal age distribution was seen only in boys. Falls were the most common injury in younger children, whereas traffic-related accidents were the most common in adolescents. With age, there was a corresponding increase in surgical treatment.

## Background

Clavicle and distal forearm fractures, primarily treated in an outpatient setting, are the most common fractures in children [1]. Although pediatric femur fractures are rare, they remain the most common traumatic orthopedic injury requiring hospitalization [2, 3]. According to Heideken et al., pediatric femur shaft fractures in Sweden in 2005 were 11.3 per 100,000. However, the frequency of this type of fracture has decreased markedly (42%) since 1987. One explanation for the decrease in femur fractures is increased safety in Swedish traffic, although a reduction in children’s physical activity may also play a role [4].

Femur fractures are more common in boys than in girls [4–6] and boys seem to have a bimodal incidence peak between the ages 2 and 3 and 16 and 19 years [5]. Unlike adults, most femur fractures in children are shaft fractures, followed by distal and then proximal fractures [6]. The injury mechanism depends on the child’s age, with younger children most likely to be injured by falls and older children and adolescents by traffic-related accidents [4–6]. In children < 1 year of age and who have not yet learned to walk, child (physical) abuse or metabolic bone disease is considered a possible cause of the femur fracture [4, 5, 7, 8].

Previous studies have reported a bimodal seasonal variation of femur fractures, with the incidence increasing during summer and late winter [4, 9]. Managing pediatric femur fractures depends on the child’s age, fracture pattern and location. Infants and toddlers can often be treated non-surgically with tractions, but spica casting has become the golden standard in this age group [10]. In contrast, children in school-age and adolescents are typically treated surgically [10, 11].

There are few nationwide register-based studies of femur fractures in children. Many studies that exist are single-center or solely focus on one part of the femur. None of the studies included stress or pathological fractures of the femur. In addition, most of the studies on femur fractures are from the last or the beginning of the twenty-first century. Therefore, we aimed to describe the modern epidemiology of femur fractures in children and adolescents aged < 16 years who were registered in the Swedish Fracture Register (SFR) from 2015 to 2018. Another aim was to investigate the association between femur fractures and age, sex, fracture pattern,  injury mechanism, seasonal variation and treatment. The main hypotheses are that (i) femur fractures are more common in boys, (ii) shaft fractures are more common than proximal or distal femur fractures and (iii) the proportion of surgical treatment increases with advancing age of the patients.

## Methods

### Data collection and study population

This observational register study was based on all pediatric femur fractures registered in the SFR. The SFR is a web-based national quality register containing detailed data on fractures of all types and includes injury mechanism, fracture localization and classification and treatment details. The treating orthopedic surgeon enters the data in the SFR. The SFR only included adult patients when it was established in April 2012 [12]. In May 2015 the register was expanded to include pediatric fractures [13].

The study population included children and adolescents < 16 years old at the time of injury. All had been diagnosed with a femur fracture. We recovered all first-time femur fractures (pathological, open and closed fractures) recorded in the SFR with a date of injury between 1 January 2015 and 31 December 2018.

### Variables

Data collected from the SFR included age at the time of injury, sex, date of injury, injury county, mechanism of injury, fracture type and segment and treatment. The children were classified by sex and age in the following groups: infancy and toddlerhood (0–3 years), preschool (4–6 years), school-age (7–12 years) and adolescence (13–15 years). The mechanism of injury was based on (ICD-10) E-codes and then categorized into seven groups: traffic accidents, falls < 1 m (m), falls > 1 m, unspecified falls, stress/pathological/spontaneous fractures, non-accidental and other accidents. All falls on the same level were categorized in the group “falls < 1 m” and all other falls were categorized in the group “falls > 1 m”. Patients injured because of physical abuse or engaging in a physical altercation were combined into the non-accidental group. Treatments were categorized into non-surgical (spica-cast and traction) and surgical (external fixation, intramedullary nailing, plate fixation, cannulated screws, sliding hip screws and unspecified).

### Statistical analysis

The statistical analyses were done using Excel (Microsoft Excel for Mac 2019 16.29.1, Microsoft Corporation, Redmond, WA) and IBM Statistical Package for the Social Sciences (SPSS version 25 for Mac, Chicago, IL). Descriptive statistics (counts, median with interquartile range [IQR] and percentage) were used to analyze age and sex distribution, mechanism of injury, seasonal variation and treatment variation. The median and IQR were used to describe nonparametric data. Logistic regression was used to estimate the odds ratio (OR) of the surgical treatment for femur fractures in relation to age, sex and location. Statistical significance was defined as *p* < 0.05.

## Results

**Fig. 1:**
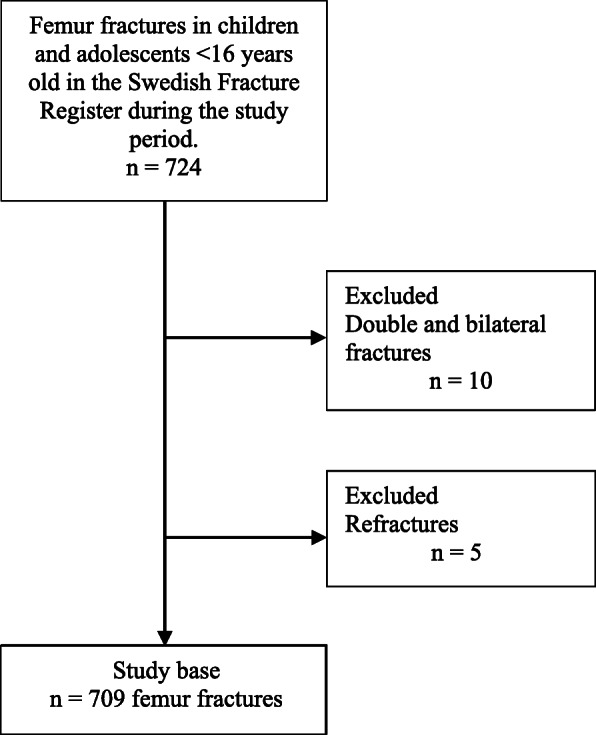
Flow chart of the study population

During the study period, 724 pediatric femur fractures were registered. If a single child had multiple fractures, those fractures with the most missing data were excluded. This was the case in 10 children: four of these children had unilateral and six had bilateral femur fractures. We also excluded refractures, which occurred in five children. Thus, the final study population was 709 femur fractures, with one fracture per child (Fig. 1).

### Age, sex and fracture type

**Fig. 2:**
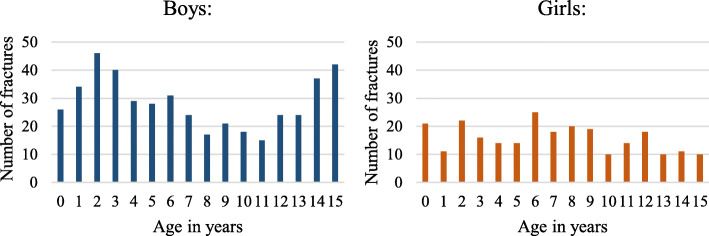
Distribution of femur fractures by age and sex

Of the 709 patients with femur fractures, 456 were boys (64%) and 253 girls (36%), yielding a boy:girl ratio of 1.8:1. A trend (*p* = 0.08) indicated that shaft fractures were slightly more common in boys, whereas proximal and distal fractures were more common in girls. The median age for a femur fracture was 6 years in boys (IQR, 3.0–12.0) and 7 years in girls (IQR 3.0–10.0) (*p* = 0.6). A bimodal fracture distribution was seen in boys, with one peak at age 2–3 years and one at 14–15 years. A similar bimodal distribution was not observed in girls (Fig. 2).

**Fig. 3:**
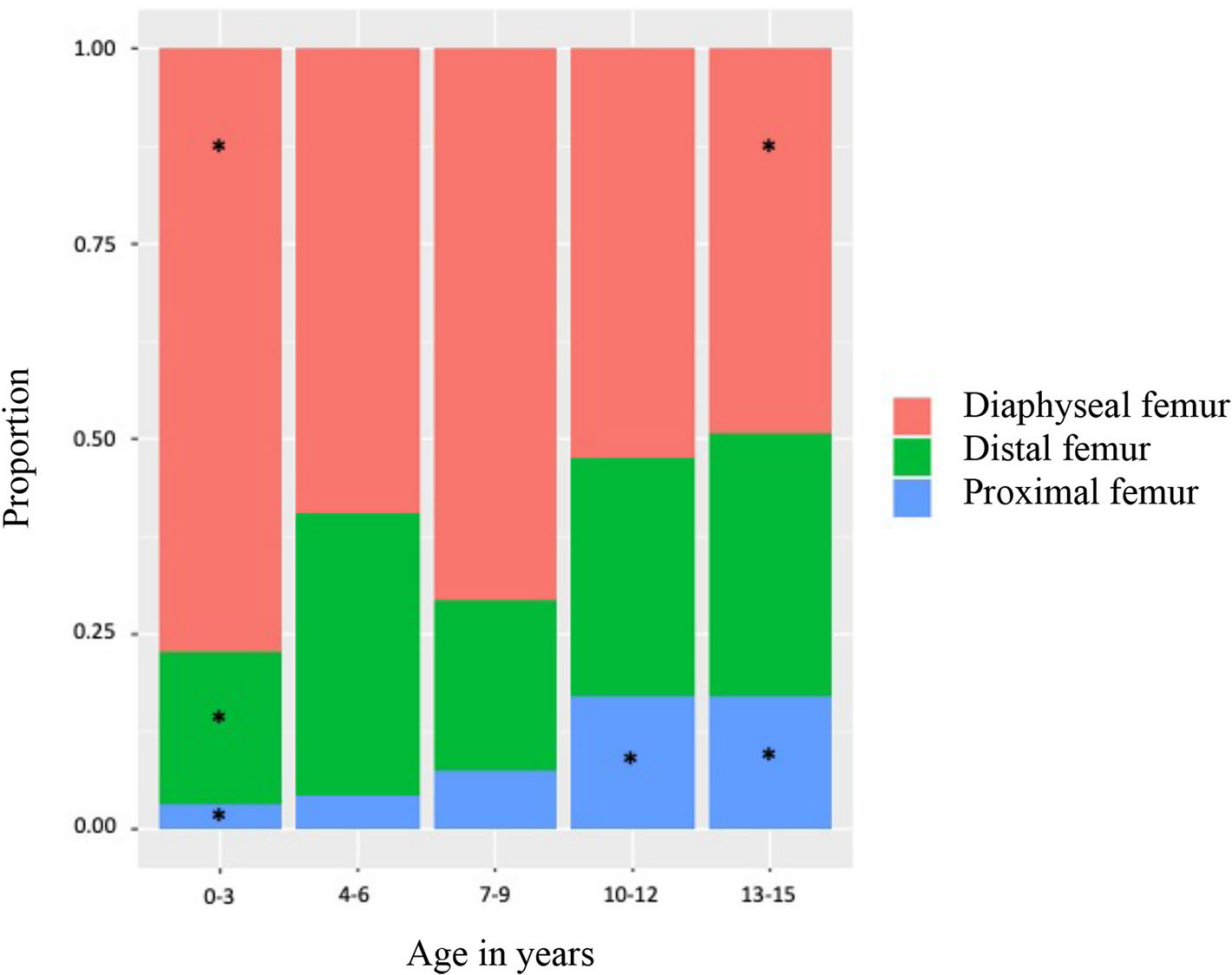
The proportion of femur fractures by age and fracture type

Of the 709 fractures, 62 were proximal (9%), 453 shaft (64%) and 194 distal (27%). Shaft fractures were the most common type of fracture in every age group, but the rate of shaft fractures varied depending on the child’s age. In the youngest age group (0 to 3 years) shaft fractures accounted for 77% of the fractures, which was significantly higher compared to the oldest age group (13–15 years), where shaft fractures accounted for 49% of the fractures. In contrast, the proportion of proximal fractures was significantly higher in the older age groups (10–12 and 13–15) years (Fig. 3).

### Mechanism of injury

Table 1 shows how the injury mechanism was contingent on the age of the child. Falls were the most common injury mechanism across all age groups, except adolescents. In children aged 0–3 years, falls from > 1 m were more common than falls from < 1 m, but in all other age groups, falls < 1 m were more frequent (Table 1).

**Table 1:** Mechanism of injury for femur fractures by the age of the child

Age (years)	0–3		4–6		7–9		10–12		13–15		Total	
Mechanism of injury	n	%	n	%	n	%	n	%	n	%	n	%
Traffic accident ^a^	12	6	14	10	24	20	25	25	51	40	126	18
Fall <1 m	70	32	47	33	45	38	34	35	40	32	236	33
Fall >1 m	79	37	44	31	29	24	14	14	6	5	172	24
Fall unspecified	16	7	9	6	3	2	6	6	4	3	38	5
Stress/ pathological/ spontaneous	2	1	5	4	3	3	3	3	7	6	20	3
Non-accidental	14	7	1	1	3	3	5	5	8	6	31	5
Other ^b^	22	10	19	14	11	9	12	12	16	6	80	11
Unspecified	1	–	2	1	1	1	–	–	2	2	6	1
**Total**	216	100	141	100	119	100	99	100	134	100	709	100

^a^Traffic accident = Accident by car, motorcycle, bicycle or accident by other vehicles

^b^Other = Horse riding and ICD code “other accident”

The rate of children injured in traffic accidents increased as age increased. Traffic accidents were the most common cause of femur fractures in the 13–15-year age group (Table 1). Some (43%) of the traffic accidents were caused by motorcycle accidents, followed by bicycle accidents (27%). More than half of the motorcycle accidents were in the age group 13–15 years and 91% of the patients were the driver and 5% the passenger. For the remaining 4%, details of the accidents were not specified in the SFR. Bicycle accidents were most prevalent in the age group of 7–9 years. Of the femur fractures, 5% were caused by non-accidental trauma and 45% of these children were 0–3 years of age. Twenty (3%) of the femur fractures were stress/pathological/spontaneous fractures and 16 of these occurred in boys. In all age groups there were more boys than girls with a stress/pathological/spontaneous fracture. Most of these fractures occurred in the age group 4–6 years (five fractures) and 13–15 (seven fractures).

Shaft fractures were the most frequent fracture type, regardless of the injury mechanism. Non-accidental trauma generated the highest percent of distal femur fractures (39%). The injury mechanism was not reported in 43 children.

### Seasonal variation

**Fig. 4:**
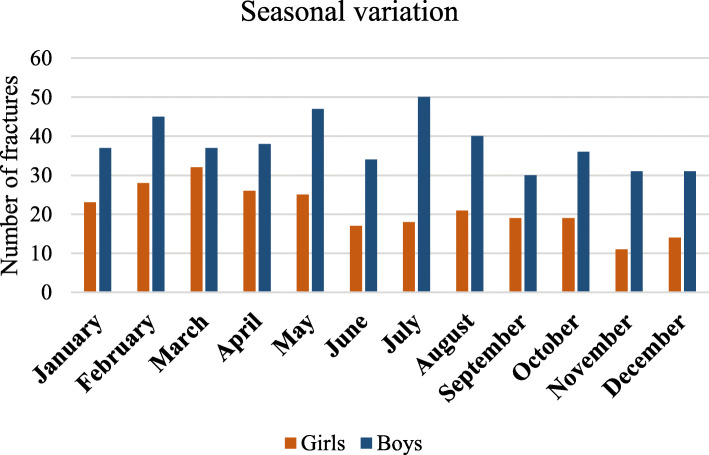
Seasonal variation of femur fracture by sex of the child

Most femur fractures occurred in February, May and July in boys and February, March and April in girls. June was the month with the lowest number of fractures in both sexes. There were always more boys than girls with a femur fracture no matter the month (Fig. 4). The number of reported fractures did not differ between the counties by month, except in some of Sweden’s mountain regions, where more femur fracture were reported in February and March than in the other months. The mechanism of injury for these fractures was mainly skiing accidents.

### Treatment

Logistic regression analysis revealed that the overall risk for surgery increased with increasing age and was highest in shaft fractures (Table 2).

**Table 2:** Risk for surgery in relation to sex, age and fracture location

Surgery risk	OR	95% confidence interval	***P***-value
Age	1.4	1.4–1.5	< 0.0001
Sex (female)	1.0	0.7–1.6	< 0.0001
**Location in reference to the proximal femur**			
Femur - Shaft	8.4	3.6–20.1	< 0.0001
Femur - Distal	0.2	0.1–0.4	0.9

#### Proximal fractures

Eighteen (29%) of the patients with proximal femur fractures were treated non-surgically (eight were in the 13–15-year group). With age, more patients were treated surgically. When surgery was performed, pin or plate fixation was applied most often to the fracture site (Table 3).

**Table 3:** Treatment of *proximal femur fractures* by the age of the child

Age (years)	0–3	4–6	7–9	10–12	13–15	Total	
Treatment	n	n	n	n	n	n	%
**Non-surgical**							
Plaster	5	–	3	2	7	17	27
Traction	–	–	–	–	1	1	2
**Surgical**							
Intramedullary nailing	–	1	–	–	–	1	2
Pin	–	–	1	7	6	14	22
Plate fixation	2	3	3	2	–	10	16
Cannulated screws	–	1	–	2	2	5	8
Sliding hip screw	–	1	1	1	3	6	10
Unspecified	–	–	1	3	4	8	13
**Total**	7	6	9	17	23	62	100

#### Shaft fractures

Non-surgical treatment was performed in 30% of the shaft fractures and intramedullary nailing in 52% (38% with flexible nails and 14% with rigid nails). Generally, older children (13–15 years) were more likely to be treated with surgical fixation than younger children (0–3 years). Intramedullary nailing shifted from flexible to rigid nails with advancing age (Table 4).

**Table 4:** Treatment of *femur shaft fractures* by the age of the child

Age (years)	0–3	4–6	7–9	10–12	13–15	Total	
Treatment	n	n	n	n	n	n	%
**Non-surgical**							
Plaster	53	5	4	–	–	62	14
Traction	63	8	–	–	–	71	16
**Surgical**							
External fixation	6	8	3	1	7	25	6
Intramedullary nailing - Rigid	2	8	8	11	36	65	14
Intramedullary nailing - Flexible	26	49	59	31	7	172	38
Plate fixation	1	1	3	2	8	15	3
Sliding screw	7	5	4	1	3	20	4
Unspecified	9	–	3	6	5	23	5
**Total**	167	84	84	52	66	453	100

### Distal fractures

In all age groups most distal fractures (69%) were treated non-surgically. Pin/cerclage fixation (11%) was the preferred method when a surgical procedure was performed. Surgical treatment increased with an increase in age (Table 5).

**Table 5:** Treatment of *distal femur fractures* by the age of the child

Age (years)	0–3	4–6	7–9	10–12	13–15	Total	
Treatment	n	n	n	n	n	n	%
**Non-surgical**							
Plaster	37	40	20	16	17	130	67
Traction	2	2	–	–	–	4	2
**Surgical**							
External fixation	–	1	1	–	4	6	3
Intramedullary nailing	–	1	–	–	–	1	1
Plate fixation	–	–	–	–	4	4	2
Pin/cerclage	–	6	4	5	7	22	11
Cannulated screw	–	–	1	4	5	10	5
Unspecified	3	1	–	5	8	17	9
**Total**	42	51	26	30	45	194	100

### Reoperations

Of the 709 patients, 124 (17.5%) underwent a registered reoperation and 18 (3%) had two reoperations. The most common reoperation was the removal of internal fixation (60%). One patient underwent 12 plastic surgeries.

## Discussion

### Main findings

In this nationwide observational register study a bimodal age distribution of femur fractures was found only in boys. Falls and traffic accidents were the most common mechanisms of injury. Most fractures were treated non-surgically. The risk for surgery increased with age and was highest in shaft fractures compared to proximal and distal fractures.

### Age, sex and fracture type

The bimodal age distribution seen in boys in this study is consistent with earlier findings [4, 5, 14]. The first peak occurs when children start to walk [4] while the second occurs during more high energy activities (e.g., motocross and rough play). These high energy activities take place more frequently in boy than in girls [15].

Our hypothesis that femur fractures are more common in boys than in girls was confirmed. The overall boy:girl ratio (1.8:1) in our study is lower compared to other studies (2.3:1 [4] and 2.6:1 [5]). One possible explanation for this discrepancy is that the studies use different age intervals. A wider age range will affect the boy:girl ratio in that adolescent boys are more often injured than adolescent girls [4, 5]. In addition, because we included pathological, stress and spontaneous fractures, comparisons are difficult to make with studies that include only traumatic injuries. However, a recent study [16] found the gap in physical activity between boys and girls to decrease, which explains the lower boy:girl ratio in femur fractures in our study.

In line with Loder et al. ‘s results, shaft fractures were the most common fracture type and thus confirmed our hypothesis [6]. We found that the proportion of shaft fractures was highest in the younger age group. In adults approximately 90% of femur fractures occur in the proximal femur [17]. Potential factors that may explain the difference in femur fracture patterns in pediatric patients compared to adult femur fractures are osteoporosis and a different manner of falling, including an increased risk of falling due to medication or impaired balance [18].

### Mechanism of injury

Falls in younger children and traffic accidents in adolescents are the two most common causes of femur fractures, an observation confirmed in our study and others [1, 4, 6]. In the youngest children (0–3 years) falls from > 1 m were more common than falls from < 1 m. One explanation can be that these children not only fall when climbing play equipment and furniture but also fall from baby changing tables or converted dressers. In contrast, older children mostly fall because of slipping or physical activity. With the child’s increasing age, the most common injury mechanism shifted from falls to traffic accidents, a finding in accord with earlier results [1, 4, 6]. In our study traffic accidents accounted for 18% of femur fractures, a percentage comparable to that reported previously [4].

Non-accidental trauma was registered in 7% of the femur fractures in children < 3 years, a percentage at variance with earlier reports [4, 6]. Loder et al. reported that 15% of femur fractures in children under the age of 2 years were due to abuse [6], whereas Heideken et al. found that abuse accounted for only 4.2% of the femur shaft fractures in children aged < 1 year [4]. Our low numbers may be because fracture registration in the SFR is conducted at the time of treatment when the exact injury mechanism is not yet clear, or the investigation of the circumstances surrounding the injury is not completed. It is difficult to compare our results to those of other studies because some studies only included shaft fractures or a different age interval. The predominance of males in the stress/pathological/spontaneous fracture group is not clear. The finding can be explained by the presence of simple or aneurysmatic bone cysts dominant in males and often located in the femur, causing pathological fracture [19, 20].

### Seasonal variation

Seasonal variations have been reported for femur fractures. For instance, Loder et al. noted an incidence peak in the summer; in contrast, Heideken et al. described a bimodal seasonal distribution, with one peak in winter/spring and another in summer [4, 6]. The differences in seasonal variation likely depend on where the study population lives. Countries (such as Sweden) with outdoor activities during winter months have shown a bimodal seasonal distribution of femur fractures [4]. In our study there were no clear peaks in the number of femur fractures during the calendar year, although a tendency for a bimodal seasonal distribution was observed. However, comparing the different counties in Sweden, a clear peak in femur fractures in Sweden’s mountain areas was noted in February and March due to skiing accidents. Traffic accidents increased during the spring and summer months, which are not surprising given that motorcycles are commonly used during the year’s warmer months.

### Treatment

In general, surgical treatment increased with age and shaft fractures had an eight-fold higher OR for being treated surgically than proximal and distal femur fractures.

#### Proximal fractures

We found a wider variety of treatment methods for proximal fractures than for shaft and distal fractures. One explanation for the difference in treatment is that proximal fracture types and severity of the fracture require different treatment methods. In addition, the surgeon’s preference plays a sizable role in the choice of treatment [21]. Broadman et al. suggest that stable and non-displaced transphyseal fractures can be treated with spica casting in children aged < 4 years [22]. All displaced fractures in children > 4 years need to be treated surgically (e.g., pins, screws or plate fixation) [21, 23].

#### Shaft fractures

As in previous studies, non-surgical treatment was the most common treatment in younger age groups and surgical treatment increased with age [11, 24]. The capacity to remodel malaligned fractures decreased as age increased, and fracture healing time increased with age. Therefore, non-surgical treatment is often less aggressive and well-tolerated in younger children [25]. However, non-surgical treatment for an adolescent would risk angular deformity, leg length discrepancy, or both. Such treatment would result in prolonged hospitalization and absence from school and thus is not optional in this age group [10, 11]. From the age of 4 years, the most common treatment method for femur shaft fractures was intramedullary nailing (external fixation and traction were only used sporadically). These results correspond to those of Heideken et al., who demonstrated that external fixation and traction decreased from 1987 to 2005, being replaced by intramedullary nailing [4].

#### Distal fractures

Little et al. propose that non-surgical treatment is an option if the fracture is stable and non-displaced [26]. Operative stabilizing internal fixation is needed to minimize the risk of angular deformity, leg length discrepancy, or both in displaced fractures [26]. We found that non-surgical procedures were most prevalent treatment practice in all age groups.

### Strengths and limitations

One strength of this study is that many hospitals report to the SFR, a nationwide register covering over 80% of Sweden’s orthopedic units (2019) [27]. Because of the SFR’s detailed reporting, many variables could be analyzed and compared to previous studies. Moreover, our study provides an updated overview of femur fractures in children and adolescents in Sweden.

Our study has several limitations. First, we could not report the incidence of femur fractures because complete registration of all femur fractures in Sweden during the study period could not be guaranteed. Missing data constitute a second limitation. For instance, treatment was not specified in 44 fractures. Moreover, surgical treatment may have better completeness in registrations than non-surgical methods as it is performed by orthopedic surgeons who are required to register the fractures. By contrast, patients treated non-surgically were sometimes treated by general pediatric surgeons not familiar with the SFR. Finally, the lack of patient-related outcome measurements for children registered in the SFR makes it impossible to determine the long-term functional impact on the patients.

## Conclusions

We found a lower ratio in femur fractures between boys and girls (1.8:1) compared to earlier studies. A bimodal age distribution was identified in boys but not in girls. Falls were the most common injury in younger children and traffic accidents the most common in adolescents. Shaft fractures were significantly overrepresented in the age group 0–3 years, whereas proximal femur fractures occurred significantly more often in children > 10 years of age. With age, there was a marked increase in surgical treatment.

## Data Availability

The dataset necessary to replicate the main findings can be obtained from the authors upon reasonable request.

## Abbreviations

SFR: Swedish Fracture Register
ICD: International Statistical Classification of Diseases and Related Health Problems 10th version
IQR: Interquartile range
OR: Odds ratio
UNS: Unspecified
